# Brazilian* Morus nigra* Attenuated Hyperglycemia, Dyslipidemia, and Prooxidant Status in Alloxan-Induced Diabetic Rats

**DOI:** 10.1155/2017/5275813

**Published:** 2017-04-16

**Authors:** Ivanildo I. da S. Júnior, Humberto de Moura Barbosa, Débora C. R. Carvalho, Ruideglan de Alencar Barros, Flávia Peixoto Albuquerque, Dionísio Henrique Amaral da Silva, Grasielly R. Souza, Nathália A. C. Souza, Larissa A. Rolim, Flaviane M. M. Silva, Glória I. B. P. Duarte, Jackson R. G. da S. Almeida, Flávio Monteiro de Oliveira Júnior, Dayane A. Gomes, Eduardo C. Lira

**Affiliations:** ^1^Department of Physiology and Pharmacology, Federal University of Pernambuco, Recife, PE, Brazil; ^2^Federal University of San Francisco Valley, Petrolina, PE, Brazil

## Abstract

*Morus nigra* has been used popularly for several proposes, including diabetic. In an attempt to support medicinal value, the acute hypoglycemic, hypolipidemic, and antioxidant effects of the ethanolic extract of* Morus nigra* (EEMn 200 or 400 mg/kg b.w.) were evaluated in normal and alloxan-induced diabetic treated for 14 days. Serum biochemical and antioxidant analysis were performed at the end of experiment. Oral glucose tolerance test was performed at 10th and 15th days. Chromatographic analysis by HPLC-DAD of EEMn was performed. Insulin was used as positive control to glycemic metabolism as well as fenofibrate to lipid metabolism. EEMn (400 mg/kg/day) reduced fasting and postprandial glycaemia, improved oral glucose tolerance, and reduced lipolysis and proteolysis in diabetic rats. EEMn decreased the blood levels of total cholesterol and increased HDL level when compared to the diabetic control rats. At higher levels, EEMn reduced triglycerides and VLDL levels in diabetic rats. Also, EEMn reduced malondialdehyde and increased the reduced glutathione levels in liver of diabetic rats. Chromatographic analysis identified the presence of the flavonoids rutin, isoquercetin, and kaempferitrin. Acute EEMn treatment reduced hyperglycemia, improved oral glucose tolerance, and minimized dyslipidemia and oxidative stress leading to a reduction in atherogenic index in alloxan-induced diabetic rats.

## 1. Introduction

Diabetes* mellitus* (DM) is the most common metabolic disorder characterized by persistent hyperglycemia, which is due to carbohydrate, protein, and lipid metabolism disturbance caused by relative or absolute deficient in insulin secretion and/or insulin action in peripheral tissues [1]. The prevalence of DM is rising steadily and becomes epidemic in modern society. Nowadays there are 415 million of diabetic patients and it is forecast to be 642 million in 2040. Currently, in Brazil there are more than 14 million diabetics and this high prevalence is associated with long-term complication including oxidative stress, dyslipidemia, microvascular, and macrovascular diseases [2].

It is well established that hyperglycemia and diabetic dyslipidemia associated with high levels of free radical and the simultaneous decline of antioxidant defense lead to several comorbidities including nephropathy, retinopathy, neuropathy, and macro- and microvascular damage [3, 4]. Intensive glycemic control, targeting glycated hemoglobin levels below 7%, is mandatory to prevent or minimize cardiovascular risk [5]. Diabetic control involves exercise, diet, and medicines. However, antidiabetic therapy has been limited by side effects. Hence there is clear need for additional alternatives to reduce the negative impact of hyperglycemia, dyslipidemia, and oxidative stress in diabetic patients [4].

World Health Organization (WHO) estimates that about three-quarters of the population mainly in the countries of Latin America, Africa, and Asia confide on plant based preparations in their traditional medicinal system for primary health care [6]. Brazil has the major genetic plant biodiversity and, due to the limited access to health assistance, almost all population has made use of medicinal plants frequently [7]. Within this context,* Morus nigra *is an exotic species popularly known as “amora miura” and is widely used by traditional community from Vale do Sao Francisco, Brazil, for treatment of DM [8]. The genus* Morus* belongs to the Moraceae family, which has 40 genera and over 1000 species widely distributed in Asia, Europe, North America, South America, and Africa [8, 9]. Species of* Morus* has been used in folk medicine as hepatoprotective, hypotensive, antipyretic, analgesic, diuretic, antidiabetic [10, 11].

Some studies carried out by our research group have demonstrated that* Morus nigra* has no toxicology effect [12] and pharmacological properties in a long-term treatment, such as hypoglycemic in normal rats [8]. These actions were reported by the presence of flavonoids, which have powerful antioxidants that are associated with their medicinal properties, including antidiabetic activity [8, 13].

In this context, the present work evaluated the in vivo effect of ethanol extract from the leaves of* Morus nigra* acutely in hyperglycemia, lipid profile, and oxidative stress in alloxan-induced diabetics in rats.

## 2. Materials and Methods

### 2.1. Plant Material and Extract Preparation

The leaves of* Morus nigra* L. were collected in Casa Nova, State of Bahia, Brazil, during October 2013. A voucher specimen (# 1764) was deposited at the Herbarium Vale do Sao Francisco (HVASF) at Federal University of San Francisco Valley. The extract was prepared by maceration of dried and powdered leaves (714 g) in 90% ethanol for three days at room temperature. The extractive solution was concentrated under vacuum and dried at 50°C. Preparation of 1 g of ethanol extract (EE) required approximately 10 g of dried leaf (yield = 9%).

### 2.2. Analysis of Phenolic Compounds by High-Performance Liquid Chromatography (HPLC)

The analysis was conducted in a liquid chromatograph (Shimadzu®) coupled to the diode array detector (DAD). The data were analyzed using Shimadzu LC solution software. Stationary phase was performed using C_18_ column (250 × 4.6 mm), 5 *μ*m particle size (Thermo Scientific®). The mobile phase consists of solvent A, water + 0.01% (v/v) trifluoroacetic acid (TFA), and solvent B, acetonitrile (ACN), according to the gradient described in Table 1, at a flow rate of 0.8 mL/min. The sample injection volume was 5 *μ*L and the detection wavelength was 340 nm. The temperature was kept constant at 37°C throughout the analysis.

### 2.3. Animals

Adult male Wistar rats (190–210 g) were housed in individual cages, under controlled conditions such as 12/12 h light/dark cycles at room temperature (22 ± 2°C) with lights on at 6:00 a.m. They were fed commercial stock diet and water ad libitum. All experimental procedures were approved in accordance with the international standards of animal protection and the ethical principles of the National Council of Animal Experimentation (CONCEA) and were approved by the Ethics Committee on Animal Use (CEUA) of the UNIVASF (protocol #0004/170912, in November 2012).

### 2.4. Diabetes Mellitus (DM) Induction

Alloxan (ALX, 40 mg/kg, Sigma®) was dissolved in saline and injected into jugular vein in rats previously fasted for 12 h. Five days after ALX injection, animals with postprandial glycaemia over 250 mg/kg were considered diabetics and included in the experimental protocol [7]. Blood samples were collected from the tail tip and glycaemia was measured by One Touch Ultra (Johnson & Johnson®) utilizing the glucose oxidase method. Nondiabetic control animals received saline injection as control.

#### 2.4.1. Experimental Procedure

Five days after ALX injection, animals were randomly divided into seven different groups: nondiabetic control (C), nondiabetic control treated with 400 mg/kg (CT400) of ethanolic extract of* M. nigra* (EEMn), diabetic (D), diabetic treated with 200 mg/kg (DT200) and 400 mg/kg (DT400) of the EEMn, diabetic treated with insulin (DI), and diabetic treated with fenofibrate (DFeno). The diabetic and nondiabetic groups received the extract or water (controls) orally by orogastric tube, once a day, during 14 days. DI received 3 U/rat of Insulin NPH, Lilly (s.c.), at 8 a.m. and 6 p.m., from day 0 to day 14. The animals were kept in metabolic cage to measured body weight, urinary volume, and fluid and food intake daily. At 10 and 15 days of experiment, blood samples for blood glucose determination were collected from the tip of the tail. At the end of experiment, the rats were euthanized and samples to biochemical analysis were collected. Heart,* soleus*,* extensor digitorum longus* (EDL), and retroperitoneal and epididymal adipose tissue were collected and weighed.

### 2.5. Biochemical Analysis

At 15th day of experiments, blood samples were obtained from abdominal aorta puncture using syringe for the determination of biochemical analysis. The levels of serum total cholesterol (TC), high density lipoprotein cholesterol (HDL-C), triglycerides (TG), and urinary urea were measured by available commercial kits (Labtest®). Urinary glucose was determined by* orto*-toluidine method [14]. The serum levels of very low density lipoprotein cholesterol (VLDL-C) were calculated using the Friedewald formula (VLDL = TG/5) [15]. Atherogenic index (AI) was determined by equation TC/HDL-C [16, 17].

### 2.6. Oral Glucose Tolerance Test (OGTT)

At 10th and 15th days of experiment, the OGTT was performed to assess the glucose tolerance in overnight fasted nondiabetic control (C), diabetic (D), and diabetic 400 mg/kg EEMn-treated animals (DT400). All animals received a load of 2.5 g of glucose/kg (v.o.) and blood glucose was measured in blood withdrawn from the tip of the tail, before load (*t* = 0) and 15, 30, 60, 90, 120, 150, 180, 210, and 240 min after glucose administration. Areas under the curve (AUC) were calculated using the trapezoidal method for comparison of the glucose utilization by tissues in normal and diabetic conditions [18].

### 2.7. Estimation of Antioxidant Activity

#### 2.7.1. Lipid Peroxidation Estimation

The lipid peroxidation was measured by thiobarbituric acid (TBAR) reaction with malondialdehyde (MDA) [19]. In brief, liver sample was dissected and immediately washed with ice-cold saline to remove blood. Tissue homogenates (20% w/v) were prepared in cold 50 mM potassium phosphate buffer (pH 7.4), using a glass-Teflon homogenizer (Ika®). TBAR reagent was added to 1.0 mL supernatant and the mixture was incubated at 95°C for 1 hour and cooled under running tap water prior to addition of 1 mL* n*-butanol. After thorough mixing, it was centrifuged at 4000 *g* (15 minutes at 4°C). The organic layer was transferred into a clear tube and absorbance was measured at 532 nm with a spectrophotometer (Thermo Scientific). The rate of lipid peroxidation was expressed as *μ* moles of MDA formed/mg protein.

#### 2.7.2. Estimation of Glutathione Peroxidase (GPx) Activity

GPx activity in liver was measured according to the method by Paglia and Valentine [1967] [20]. The reaction mixture consists of 0.2 mL, 0.8 mM EDTA; 0.1 mL, 10 mM sodium azide; 0.1 mL, 2.5 mM H_2_O_2_; 0.2 mL, 5 mM GSH; 0.4 mL 0.4 mM phosphate buffer (pH 7.0); and 0.2 mL homogenate which were incubated at 37°C for 10 minutes. The reaction was arrested by the addition of 0.5 mL of 10% TCA and was centrifuged at 2000 rpm. 3.0 mL of 0.3 M disodium hydrogen phosphate and 1.0 mL of DTNB were added to the supernatant and the color changes were read immediately by using a spectrophotometer (Thermo Scientific) at 420 nm. GPx activity levels were expressed as *μ* moles of formed/mg of protein.

### 2.8. Statistical Analysis

Data were expressed as mean ± SEM. The one-way analysis of variance (ANOVA) was employed to analyze the data between treated groups and their respective control groups (diabetic and nondiabetic) followed by Tukey test. Two-way ANOVA was employed to analyze OGTT data followed when necessary by Bartlett's test. Differences were considered significant at *P* < 0.05.

## 3. Results

### 3.1. High-Performance Liquid Chromatograph Analysis

**Figure 1:**
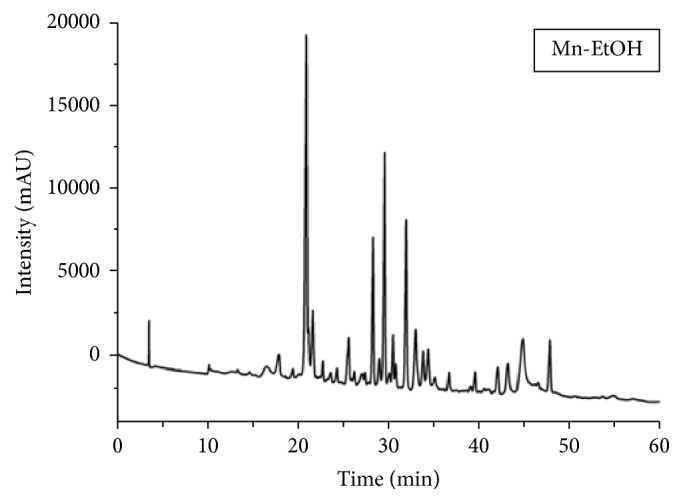
Phenolic compounds profile of the EEMn.

The profile of phenolic compounds of ethanolic extract of* Morus nigra* (EEMn) leaves was analyzed by HPLC (Figure 1 and Table 2). Based on the chromatograms expressed in Figure 1, it was possible to identify the presence of the flavonoids rutin, isoquercetin, and kaempferitrin. These compounds were identified by comparison with the parameters retention time and absorption bands in the ultraviolet spectra of the standards compounds (Table 2).

### 3.2. Antidiabetic Effect of* Morus nigra* Leaves

**Figure 2:**
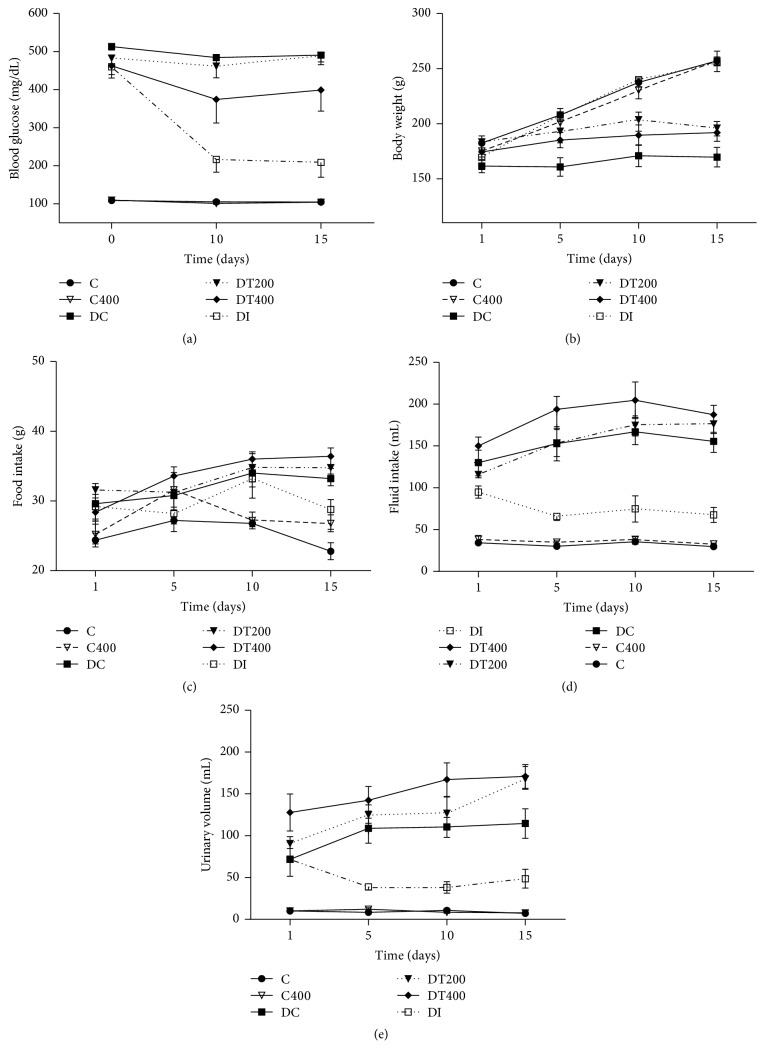
Effect of the EEMn on (a) postprandial glucose, body weight (b), food intake (c), fluid intake (d), and urinary volume (e) of CT400, nondiabetic control rats treated with 400 mg/Kg of EEMn; DC, diabetic control; DT200, diabetic rats treated with 200 mg/Kg of EEMn; DT400, diabetic rats treated with 400 mg/Kg of EEMn; DI, diabetic rat treated with insulin (3 U/rat). Each point represents the means ± SEM of 6-7 animals.

The dose administered of ALX induced a diabetic moderate to severe degree, increasing ~6-fold in the fasting (467.0 ± 47.9 versus 72.6 ± 2.0 mg/dL) and postprandial (513.0 ± 9.1 versus 108.8 ± 4.2 mg/dL, ~5 fold) glycaemia as compared to control rats before treatment (day 0). Oral administration of EEMn showed a significant reduction in fasting glycaemia (467.0 ± 47.9 versus 315.6 ± 23.5 mg/dL) at the 15th day of treatment and postprandial glycaemia at 10th and 15th of treatment (Figure 2(a)). Furthermore, insulin treatment of diabetic rats showed a significant reduction in blood glucose level at the 10th and 15th of treatment. In the present study, diabetic rats develop diabetic symptoms such as lower weight gain, excessive food intake, polydipsia, polyuria, glycosuria, and excessive urinary urea excretion. Also, lipolysis and proteolysis are significantly increased. As shown in Table 3, EEMn (400 mg/kg/day) treatment improved weight gain (80%), increased epididymal (~5-fold), and retroperitoneal (~8-fold) adipose tissue as well as* soleus* muscle weight (50%) in diabetic rats. No significant change in heart and liver weight was observed among the groups studied (Table 3).

**Figure 3:**
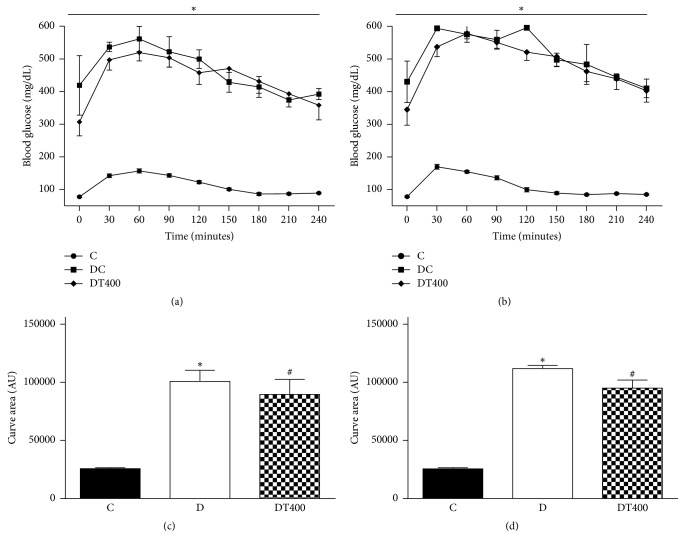
Effect of the EEMn on oral glucose at 10th (a) and 15th (b) day of experiment. C, nondiabetic control; DC, diabetic control; DT400, diabetic rats treated with 400 mg/Kg of EEMn. Area under curve (AUC) was calculated to determine glucose tolerance at 10th (c) and 15th (d) of treatments. Each point represents the means ± SEM of 7-8 animals. ^*∗*^*P* < 0.05 versus C; ^#^*P* < 0.05 versus D.

Analysis of OGTT at 10th and 15th showed that the rat of diabetic group had a significant elevation in blood glucose level throughout the total measurement period with respect to normal control (Figure 3); moreover, it did come back to initial value at the end of period tested. EEMn treatment (400 mg/kg/day) induced a significant reduction in AUC compared to diabetic control group at 10th and 15th day.

### 3.3. Hypolipidemic Properties of* Morus nigra* Leaves

**Figure 4:**
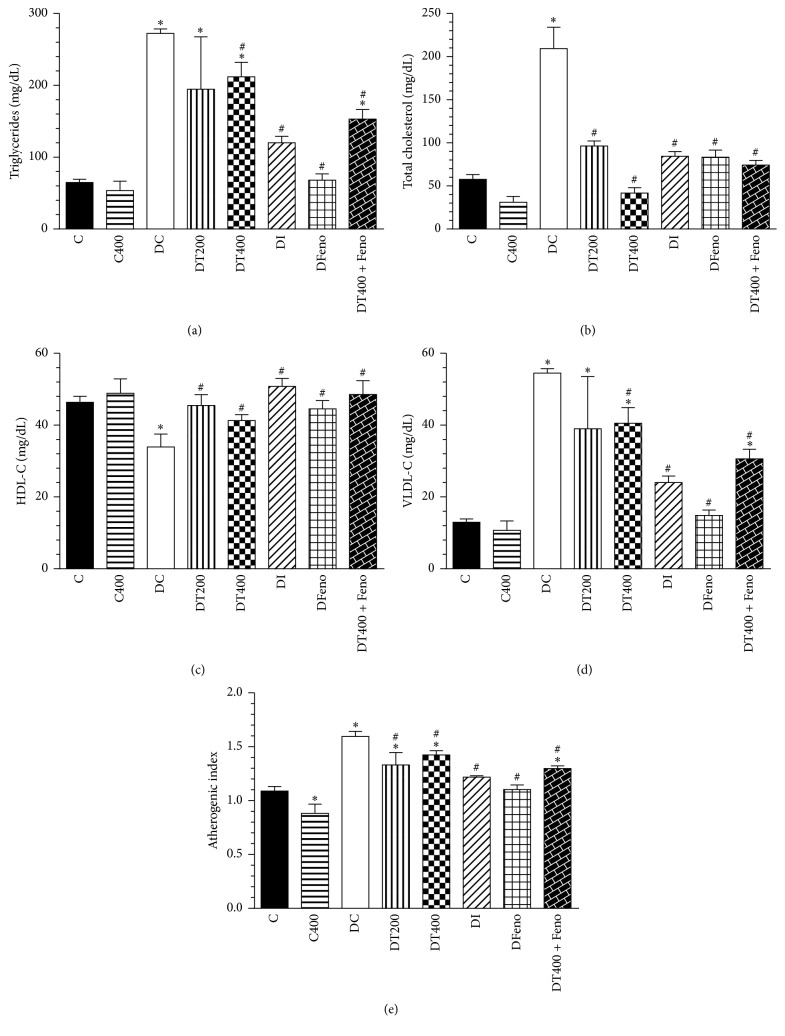
Effects of the EEMn on lipid profile and atherogenic index after 14 days of experiment. C, nondiabetic control; CT400, nondiabetic control rats treated with 400 mg/Kg of EEMn; DC, diabetic control; DT200, diabetic rats treated with 200 mg/Kg of EEMn; DT400, diabetic rats treated with 400 mg/Kg of EEMn; DI, diabetic rat treated with insulin (3 U/rat); DFeno, diabetic rat treated with fenofibrate (150 mg/Kg). Each point represents the means ± SEM of 6–8 animals. ^*∗*^*P* < 0.05 versus C; ^#^*P* < 0.05 versus DC.

Diabetic rats showed a significant increase in the blood levels of TG, total cholesterol, VLDL-C, and atherogenic index (Figure 4). On the other hand, HDL-C was significantly reduced relative to control rats. Insulin treatment decreased the blood levels of triglycerides, total cholesterol, VLDL-C, and atherogenic index. As seen in Figure 4, EEMn treatment at both doses decreased the blood levels of total cholesterol (~80%) and atherogenic index (10%) and increased HDL-C (15%) level when compared to the diabetic control rats. In addition, at higher levels, EEMn (400 mg/kg/day) treatment reduced TG (20%) and VLDL-C (30%) levels in diabetic rats. The minor dose was more efficient in increased HDL levels (30%) than higher dose (15%) of EEMn. Fenofibrate, peroxisome proliferator-activated receptors alpha (PPAR*α*), improved the lipid disorder, including reduction in VLDL (~70%), TC (60%), and TG (70%), and increased in HDL-C (25%) levels in diabetic rats. Also, fenofibrate reduced the atherogenic index (~30%) in diabetic rats.

### 3.4. Antioxidant Properties of* Morus nigra* Leaves

**Figure 5:**
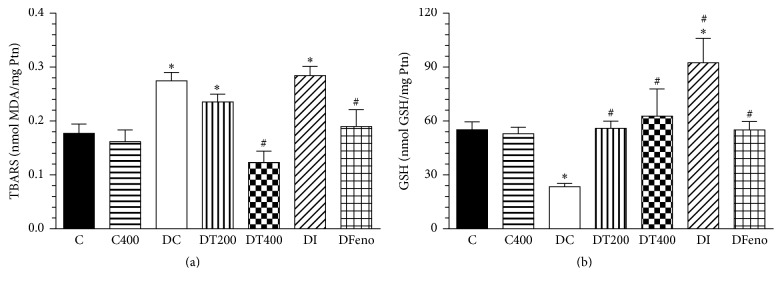
Effects of the EEMn on malondialdehyde (MDA) (a) and reduced glutathione (GSH) (b) levels after 14 days of experiment. C, nondiabetic control; CT400, nondiabetic control rats treated with 400 mg/Kg of EEMn; DC, diabetic control; DT200, diabetic rats treated with 200 mg/Kg of EEMn; DT400, diabetic rats treated with 400 mg/Kg of EEMn; DI, diabetic rat treated with insulin (3 U/rat); DFeno, diabetic rat treated with fenofibrate (150 mg/Kg). Ptn, protein. Each point represents the means ± SEM of 6–8 animals. ^*∗*^*P* < 0.05 versus C; ^#^*P* < 0.05 versus DC.

Furthermore, the antioxidant property of EEMn was evaluated using MDA and GSH levels. As shown in Figure 5, short-term treatment with EEMn reduced MDA and increased the reduced GSH levels in liver of diabetic rats but had no effect in control animals.

## 4. Discussion

Energy metabolism damage and increased oxidative stress are a common pathogenic factor leading to diabetic comorbid. In addition, diabetic dyslipidemia is a pivotal and general risk factor to heart disease, leading to increase of morbidity and mortality worldwide in economic active population [21]. It is well known that medicinal plants are widely used to treat diabetes due to their effectiveness, safety, and acceptability. The present study highlighted the short-term treatment with EEMn reduced hyperglycemia, improved oral glucose tolerance, and minimized dyslipidemia and oxidative stress leading to a reduction in atherogenic index in alloxan-induced diabetic rats. Thus, our data suggest that use of* Morus nigra* leaves acutely may be helpful in DM treatment and their comorbidities.

The extracts were in accordance with literature data when comparing the results of our study with those of previously published studies [22, 23]. The variation of quantitative phenolic compounds in the extract depends on many factors, such as degree of maturity at harvest, genetic differences, and environmental conditions. In a study by Zhang et al. [2011] [13] in order to verify a possible hypoglycemic activity, isoquercetin in diabetic mice KK-A^y^ has been frequently used as an animal model for noninsulin-dependent diabetes. The results showed that all isoquercetin-treated KK-A^y^ mice showed decreases in blood glucose levels at 60 and 120 min compared with the control KK-A^y^ mice. Hunyadi et al. [2012] [24] evaluated the antidiabetic activity of the ethanol extract of* Morus alba* using the model hypoglycaemic activity on nonneonatal streptozotocin induced diabetic rats. They found that after 11 days of treatment, the extract showed a significant, dose-related antidiabetic activity on this in vivo model of type II diabetes.

Alloxan is a selective *β*-cytotoxic agent which has been widely used to mimic diabetic pathology in rodents [25]. It is a uric acid derivate that causes dramatically reduction in serum insulin for destroying *β*-cells of the islets of Langerhans [26]. It has been known that insulin-induced suppression of endogenous glucose production, which is an essential mechanism to normoglycemia, is affected in DM chronically [27]. Here, short-term use of CEEMn (400 mg/Kg/day) had antihyperglycemic effect which linked to reduction in fasting and postprandial blood glucose in diabetic group. This glycaemia reduction may be linked to inhibition of hepatic gluconeogenesis suggested by least substrate available to glucose production from protein and lipid catabolism (reduction in urinary urea in CEEMn treated diabetic rats). Lipolysis and proteolysis are involved in the body weight decreasing of diabetic rats. Improved glycemic levels can be as a result of higher glucose utilization by cells, thereby preserving adipose and muscular tissue and contributing to body weight recovery in diabetic treated animals [7]. These results revealed that the EEMn induced an increase in glucose utilization and glucose tolerance through the body tissues of diabetic rats. These effects associated with reduction in fasting blood glucose may be due to the raise in glucose utilization by peripheral tissues or inhibition of hepatic glucose output.

Considering theses data, the antihyperglycemic effect of short-term treatment of EEMn may be due to the stimulation of insulin release from beta cells or lowered peripheral resistance to insulin. It this way, Araujo et al. [2015] [28] showed that long-term use of* M. nigra* extract raised insulinemia in diabetic rats. Insulin-induced suppression of endogenous glucose production, which is an essential mechanism to normoglycemia, is affected in DM chronically [27]. The hypoglycemic effect could be explained by extrahepatic or pancreatic mechanisms, such as an increase in renal glucose excretion (glycosuria), which is rejected by short-term treatment where reduction in glycosuria was detected in EEMn-treated diabetic animals.

It is well known that insulin deficiency in diabetes leads to atherogenic lipid profile typically marked by lipids accumulation such as TG, LDL-C, and reduced HDL-C levels, contributing to raising the cardiovascular risk [29]. Here, EEMn at high dose attenuated diabetic dyslipidemia and improved atherogenic profile. Similar effects were found in diabetic pregnant female treated with aqueous extract of* Morus nigra *[11]. The effect of EEMn in lipid profile might be attributable to the improvement in insulin action [9] besides other lipid-lowing mechanisms, such as nuclear receptor peroxisome proliferator-activated receptors alpha (PPAR*α*s) activation or modulation of cholesterol synthesis. Here, EEMn reduced hyperglycemia and spared adipose and skeletal muscle mass, which could explain, at least in part, insulin effect. There was a similar effect between fenofibrate and EEMn, treatment which could explain, at least in part, the EEMn lipid lowering effect throughout PPAR*α* activation. Further studies are needed to prove this hypothesis.

It is well known that hyperglycemia is the most important event to development of oxidative stress, but hyperlipidemia is also implicated in excessive ROS production and reduced antioxidant defense system, leading to oxidation of macromolecules such as lipids and DNA damage, contributing to apoptosis and necrosis [17]. Overproduction of reactive oxygen species such as hydrogen peroxide and molecular oxygen modulates biological function of all biomolecule, being lipids target to oxidation to generate malondialdehyde (MDA), a marker of lipid damage [30, 31]. Reduced glutathione is the most important redox buffer of the cell.

Therefore, MDA and reduced GSH represent the index of lipid peroxidation and protective cellular system against oxidative stress. These data confirm the oxidative imbalance involvement in the metabolic degenerating in DM, as well as the in vivo antioxidant effect of EEMn in diabetic rats.

This antioxidant effect of species of the genus* Morus* has been demonstrated both in vitro and in vivo [32–34]. Previous works have shown that aqueous extract of* M. nigra* leaves raised SOD activity and reduced oxidative stress in normal and hyperglycemic rats [11, 28].

Several classes of phytochemicals have antioxidant property, among them flavonoids which are the most promising agents for treatment of oxidative stress-related disease [35]. In addition, it has been demonstrated that rutin improves glycemic levels and lipid profile in rats fed with hypercaloric diet and chronic ethanol consumption [36]. The improvement in hyperglycemia, lipid profile, and oxidative stress in diabetic rats treated by EEMn can be associated with rutin, isoquercetin, and kaempferitrin presence and their antioxidant effects. Other phytochemical compounds can not be excluded, once that nuclear magnetic resonance (NMR) technique has demonstrated other molecules present in* M. nigra*, such as triterpenes [37], among them betulinic acid, which modulates antioxidant effect [38].

## 5. Conclusion

In summary, this study confirms the ethnopharmacology use of* M. nigra* as antidiabetic potential acutely. In addition, these results highlight that EEMn treatment of diabetic rats acutely may prevent diabetic comorbidities, which makes Brazilian* M. nigra *important source of new molecular to treatment of DM.

## Figures and Tables

**Table 1:** System gradient used for HPLC analysis.

	Time (min)	Solvent A (%)	Solvent B (%)
Linear gradient	0–40	100–60	0–40
Isocratic	40–50	60	40
Linear gradient	50–60	60–100	40–0

**Table 2:** Retention times (RT) and maximum absorption (*λ*max) of the phenolic standards and their correlation with the compounds of *Morus nigra* (EEMn) leaves (peaks).

Peaks	Phenolic compounds	RT (min)	*λ*max (nm)
A	Rutin	28.61	256, 353
B	Isoquercetin	29.75	260, 354
C	Kaempferitrin	31.96	267, 346

**Table 3:** Effects of EEMn on body weight gain, urinary urea, urinary glucose, heart, liver, epididymal and retroperitoneal adipose tissue, muscles *soleus*, and *extensor digitorum longus* (EDL) of nondiabetic control (C), nondiabetic control 400 mg/Kg extract treated (CT400), diabetic control (DC), diabetic 200 mg/kg (DT200), and 400 mg/Kg (DT400) extract treated and diabetic treated with insulin (3 U/rat, DI).

Groups	Body weight gain (g)	Urinary urea (g/24 h)	Urinary glucose (mg)	Heart (mg/100 g)	Liver (g/100 g)	RAT (mg/100 g)	EAT (mg/100 g)	*Soleus* (mg/100 g)	EDL (mg/100 g)
C	80.5 ± 4.0	0.42 ± 0.03		349.2 ± 4,6	4.2 ± 0.27	607.0 ± 34.9	682.0 ± 24.3	11.4 ± 2.14	12.29 ± 1.22
CT400	74.7 ± 5.0	0.41 ± 0.03	—	363.5 ± 7.7	4.5 ± 0.12	940.0 ± 154.7	955.6 ± 76.7^*∗*^	11.2 ± 1.16	11.59 ± 0.39
DC	16.3 ± 4.4^*∗*^	1.89 ± 0.14^*∗*^	—	372.8 ± 14.0	4.4 ± 0.21	37.3 ± 25.7^*∗*^	120.9 ± 37.8^*∗*^	9.3 ± 1.25^*∗*^	9.72 ± 1.70^*∗*^
DT200	12.4 ± 6.8	1.19 ± 0.09^#^	41.88 ± 3.82^*∗*^	328.4 ± 3.1	4.2 ± 0.15	54.2 ± 29.8	285.0 ± 62.4	10.2 ± 1.28	10.59 ± 2.53
DT400	29.0 ± 5.4^#^	1.03 ± 0.04^#^	18.65 ± 1.10^#^	390.7 ± 26.1	4.2 ± 0.13	326.0 ± 79.7^#^	642.0 ± 48.0^#^	13.8 ± 2.97^#^	10.30 ± 1.28
DI	83.2 ± 8.0^#^	0.81 ± 0.13^#^	13.57 ± 0.15^#^	357.9 ± 16.5	9.8 ± 0.27	288.0 ± 16.0^#^	229.0 ± 32.0^#^	10.23 ± 1.18	11.40 ± 1.45^#^

Each point represents the means ± SEM of 5–7 animals. ^*∗*^*P* < 0.05 versus C; ^#^*P* < 0.05 versus DC.
